# Species distribution models for the eastern blacklegged tick, *Ixodes scapularis*, and the Lyme disease pathogen, *Borrelia burgdorferi*, in Ontario, Canada

**DOI:** 10.1371/journal.pone.0238126

**Published:** 2020-09-11

**Authors:** Andreea M. Slatculescu, Katie M. Clow, Roman McKay, Benoit Talbot, James J. Logan, Charles R. Thickstun, Claire M. Jardine, Nicholas H. Ogden, Anders J. Knudby, Manisha A. Kulkarni

**Affiliations:** 1 School of Epidemiology and Public Health, University of Ottawa, Ottawa, Ontario, Canada; 2 Department of Population Medicine, Ontario Veterinary College, University of Guelph, Guelph, Ontario, Canada; 3 Department of Pathobiology, Ontario Veterinary College, University of Guelph, Guelph, Ontario, Canada; 4 Canadian Wildlife Health Cooperative, Ontario Veterinary College, University of Guelph, Guelph, Ontario, Canada; 5 Public Health Risk Sciences Division, National Microbiology Laboratory, Public Health Agency of Canada, Saint-Hyacinthe, Quebec, Canada; 6 Department of Geography, Environment, and Geomatics, University of Ottawa, Ottawa, Ontario, Canada; University of North Dakota School of Medicine and Health Sciences, UNITED STATES

## Abstract

The blacklegged tick, *Ixodes scapularis*, is established in several regions of Ontario, Canada, and continues to spread into new geographic areas across the province at a rapid rate. This poses a significant public health risk since *I*. *scapularis* transmits the Lyme disease-causing bacterium, *Borrelia burgdorferi*, and other pathogens of potential public health concern. The objective of this study was to develop species distribution models for *I*. *scapularis* and *B*. *burgdorferi* to predict and compare the potential distributions of the tick vector and the Lyme disease pathogen as well as the ecological factors most important for species establishment. Ticks were collected via tick dragging at 120 sites across southern, central, and eastern Ontario between 2015 and 2018 and tested for tick-borne pathogens. A maximum entropy (Maxent) approach was used to model the potential distributions of *I*. *scapularis* and *B*. *burgdorferi*. Two independent datasets derived from tick dragging at 25 new sites in 2019 and ticks submitted by the public to local health units between 2015 and 2017 were used to validate the predictive accuracy of the models. The model for *I*. *scapularis* showed high suitability for blacklegged ticks in eastern Ontario and some regions along the shorelines of the Great Lakes, and moderate suitability near Algonquin Provincial Park and the Georgian Bay with good predictive accuracy (tick dragging 2019: AUC = 0.898; ticks from public: AUC = 0.727). The model for *B*. *burgdorferi* showed a similar predicted distribution but was more constrained to eastern Ontario, particularly between Ottawa and Kingston, and along Lake Ontario, with similarly good predictive accuracy (tick dragging 2019: AUC = 0.958; ticks from public: AUC = 0.863. The ecological variables most important for predicting the distributions of *I*. *scapularis* and *B*. *burgdorferi* included elevation, distance to deciduous and coniferous forest, proportions of agricultural land, water, and infrastructure, mean summer/spring temperature, and cumulative annual degree days above 0°C. Our study presents a novel application of species distribution modelling for *I*. *scapularis* and *B*. *burgdorferi* in Ontario, Canada, and provides an up to date projection of their potential distributions for public health knowledge users.

## Introduction

Lyme disease is a tick-borne illness caused by the *Borrelia burgdorferi* sensu lato bacterial complex [1]. In eastern North America, the bacterium is transmitted to humans through the bite of the blacklegged tick, *Ixodes scapularis* [2–4]. Northward expansion of *I*. *scapularis* populations, at least in part attributable to climate change, is driving the emergence of Lyme disease in new regions and increasing the number of people at risk, particularly in Canada [5–7]. From 2009–2017, approximately 6,000 cases of Lyme disease were reported in Canada and the national annual incidence is estimated at 2.7 cases per 100,000 population [8]. However, in some locations, the incidence of Lyme disease is substantially higher, with public health units in the province of Ontario reporting estimates of 18 cases per 100,000 for the City of Ottawa (OTT), 87 cases per 100,000 for Kingston, Frontenac, Lennox and Addington (KFL), and almost 130 cases per 100,000 for Leeds-Grenville and Lanark District (LGL) in 2017 [9]. In some locations in Canada, Lyme disease poses a very significant public health risk, reaching similarly high incidence (≥ 10 cases per 100,000 population per year) as observed in states in the northeastern United States [10].

The risk of contracting Lyme disease depends on several factors including human activity and behaviours as well as the density of *B*. *burgdorferi* infected ticks in the environment, which is determined by both the abundance of *I*. *scapularis* and the proportion of ticks infected with *B*. *burgdorferi* [11–14]. The most common method used for estimating risk of exposure to *B*. *burgdorferi* is the identification of *I*. *scapularis* populations in the environment using various surveillance methods [15]. In Canada, the spatial distribution of *I*. *scapularis* ticks has been studied via passive tick surveillance programs that rely on tick submissions from the public and healthcare providers, and by active tick surveillance that utilizes mainly drag sampling techniques to sample the environment for questing ticks [16]. On a broader geographic scale, province-wide surveillance studies have shown that the distribution of *I*. *scapularis* populations is affected by climatic factors, which play an important role in the occurrence and abundance of arthropod vectors and in delimiting the potential range of the vector [5–7, 17, 18]. At a local scale, site-level surveillance studies have shown that the distribution of *I*. *scapularis* is also affected by ecological factors like understory density, presence of shrubs, dominant tree type, canopy cover, proportion of forested land and forest fragmentation that are integral to the life cycle of the ticks [19–22]. These factors also contribute to an adequate habitat for mammalian hosts such as white-footed mice (*Peromyscus leucopus*) and white-tailed deer (*Odocoileus virginianus*) that are integral to the developmental and reproductive cycle of the ticks, which are obligate ectoparasites [20]. Ecological and climatic factors are often inter-related, and both contribute to the establishment of tick populations at different spatial scales. This has led to the use and development of complex modelling strategies to identify the geographic distribution of ticks or to make predictions about potential habitat suitability based on a wide variety of earth observation data [6, 21, 23, 24].

Species distribution models (SDM) are a variety of statistical models that relate species distribution data (e.g. occurrence or abundance at known locations) to information on the environmental or spatial characteristics of those locations [25]. SDMs mainly differ in the type of species data they use, with some requiring absence/presence or abundance data (e.g. generalized linear and additive models, GLM and GAM; random forests; boosted regression trees, BRT) while others rely solely on presence-only data (e.g. maximum entropy models, Maxent; genetic algorithm for rule set production, GARP) [25]. Of these, Maxent is one of the most frequently used SDMs and has shown consistently high performance compared to other models [26, 27]. In this study, we used Maxent to predict the distribution of *I*. *scapularis* and *B*. *burgdorferi* in south-eastern Ontario, and to identify factors that contribute to the establishment of the pathogen, in order to more accurately estimate environmental risk for Lyme disease. Our models provide the most up-to-date environmental risk maps for Lyme disease in the province of Ontario, Canada, where the number of human Lyme disease cases is growing annually as the tick increases its range.

## Materials and methods

### Species occurrence data and study area

**Fig 1:**
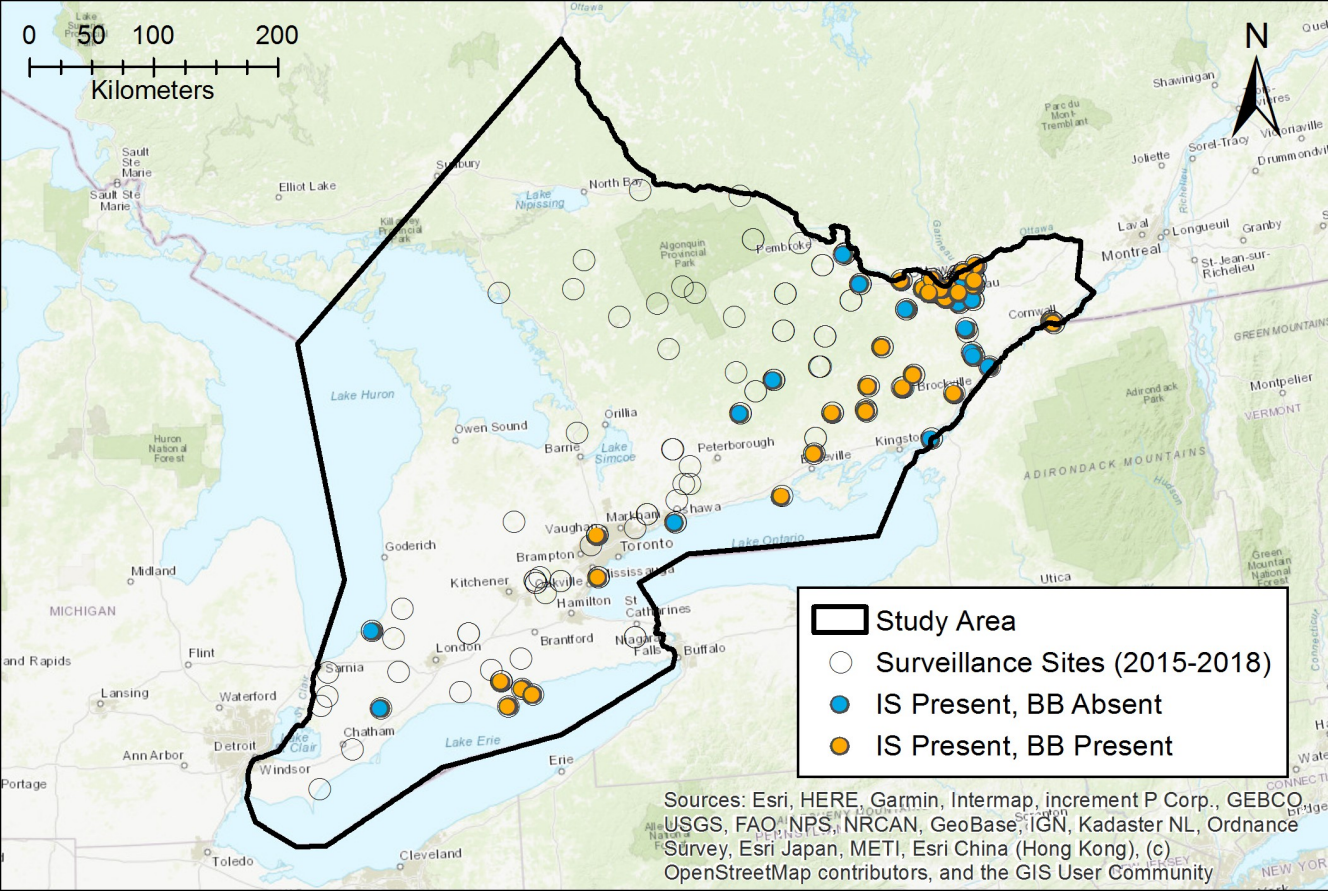
Study area and tick field sampling locations conducted between 2015–2018. Open circles show sites sampled for ticks, blue circles indicate sites where *Ixodes scapularis* (IS) ticks were found, and orange circles show sites where *Borrelia burgdorferi* (BB) was detected through molecular testing.

Occurrence data were compiled from field collection of immature and adult *I*. *scapularis* ticks made by the University of Guelph and the University of Ottawa from 2015–2018 in Ontario, Canada. We received authorization from Ontario Parks, the City of Ottawa, the National Capital Commission, Queen’s University Biological Station, Upper Canada Migratory Bird Sanctuary as well as relevant regional conservation authorities (e.g. Grey Sauble, Mississippi Valley, Cataraqui). Both field teams employed a standard field dragging protocol in which a one-meter squared white flannel cloth sheet was dragged along surface vegetation and the forest floor for three person-hours in a given site [28, 29]. The drag sheets and surveyors’ clothes were checked for ticks every 3 minutes (University of Guelph) or every 50 meters with step counts adjusted for each individual's walking pace (University of Ottawa) [28, 29]. Latitude/longitude coordinates were recorded for each sample location using a hand-held GPS. All selected sites were visited during the summer months (May to August) to capture the peak season for questing nymphs. In eastern Canada, the density of questing adult ticks often peaks in the spring and fall, while the density of questing nymphs peaks in the summer [5]. However, various sites were revisited multiple times during the summer and fall to address other research questions. For this study, we used all sampling data available to maximize our ability to detect ticks. Locations to be sampled were selected by each University independently as described in earlier work, and included sites both known, suspected, or broadly suitable for *I*. *scapularis* as well as control sites, negative sites, or unsuitable *I*. *scapularis* sites distributed across three ecoregions (5E, 6E, 7E) of Ontario and within urban, suburban, and rural regions [24, 28–30]. A total of 120 sites were sampled between 2015 and 2018 across southern, central, and eastern Ontario (Fig 1). *Ixodes scapularis* ticks were found at 52 locations (Fig 1).

All larval, nymphal, and adult ticks found in the field were collected in specimen tubes and sent to two laboratories for analysis. Ticks collected by the University of Guelph were shipped in 70% ethanol to the National Microbiology Laboratory (Public Health Agency of Canada, Winnipeg, Manitoba, Canada) for species identification. All adult and nymphal *I*. *scapularis* ticks were further tested for the presence of *B*. *burgdorferi*, *Borrelia miyamotoi*, *Anaplasma phagocytophilum*, and *Babesia microti* by real-time PCR [31, 32]. Ticks collected by the University of Ottawa were identified using standard taxonomic keys and tested for the same tick-borne pathogens within the university [29, 32–36]. Prior to testing, real-time PCR assays established at the University of Ottawa were validated using a panel of test samples provided by the NML to ensure comparable results between the two laboratories. Out of the 52 locations in Ontario where *I*. *scapularis* ticks were found, 33 sites had at least one tick positive for *B*. *burgdorferi* (Fig 1). The prevalence of the other tick-borne pathogens is low in Canada and there was insufficient occurrence data for other pathogen species to be included in this study.

### Earth observation data and environmental variables

Satellite remote sensing data pertaining to climate, land cover / land use, and elevation were selected to identify areas conducive to the establishment of *I*. *scapularis* and *B*. *burgdorferi*. All grid data was projected into the NAD83 Lambert Conformal Conic projection and resampled at 100-meter resolution.

For land cover variables, we used the Southern Ontario Land Resource Information System version 3.0 (SOLRISv3.0), which is based on Landsat-7 ETM+ satellite imagery captured between 2000–2015 and classified into 30 land cover types found in Ontario at 15-meter pixel resolution. We supplemented this with the Ontario Land Cover Data Base 2000 (OLCDB2000), which is based on Landsat-7 ETM+ satellite imagery captured between 1999–2002 at 25-meter pixel resolution, for UTM zones 17 and 18. All land cover datasets were obtained from the Ministry of Natural Resources and Forestry’s open data portal (https://geohub.lio.gov.on.ca). We used ArcMap 10.5.1 (ESRI, Redlands, CA) to mosaic images from SOLRISv3.0 and OLCDB2000 to create a complete grid for our study area, with precedence given to the most recent land cover data obtained from SOLRISv3.0 for any overlapping grid cells. This composite land cover raster was resampled to 100-meter resolution then reclassified into 10 land cover types that dominate southern, central, and eastern Ontario (S1 Table). To derive our explanatory variables, for each of these 10 land cover types we calculated 1) the proportion of each land cover type within a 1000 meter circular buffer of each grid cell, and 2) the distance of each grid cell to the land cover type, yielding a total of 20 land cover variables (S2 Table).

For climate variables, we used the Canada-wide long-term climate averages for 1981–2010 obtained from Natural Resources Canada [37]. This dataset consists of 19 bioclimatic parameters derived from temperature and precipitation records at 5 km resolution. Additionally, we obtained grid data for annual cumulative degree days above 0°C (DD > 0°C), calculated as the sum of all days of the year with mean surface temperatures > 0°C as reported by climate stations within a 5 km grid. DD > 0°C is often used as the main climatic indicator for tick establishment particularly when used in models of future climate projections [5–7].

For the elevation variable, we used the Ontario Digital Elevation Model (ODEM), which is a 3-dimensional raster dataset that captures terrain elevations for the province of Ontario at a resolution of 30 meters (available at: https://geohub.lio.gov.on.ca). In total, we derived 41 environmental variables for land cover, climate, and elevation for our *I*. *scapularis* and *B*. *burgdorferi* species distribution models; all rasters were resampled to 100-meter resolution. The complete list of the derived explanatory variables is shown in S2 Table.

### Model development

We used Maxent v.3.4.0 to model the potential distribution of suitable habitat for *I*. *scapularis* and *B*. *burgdorferi* in south-eastern Ontario. Maxent uses species presence data and randomly selected pseudo-absences (i.e. background points) to generate a probability distribution across a landscape, often conceptualized as habitat suitability or an approximation of the species’ niche, constrained within a set of environmental parameters at presence locations [38]. As such, Maxent functions under the assumption that species presence points represent an unbiased sample from the species’ realized niche [38]. Thus, we used species presence data derived from active field dragging because it is the most accurate method for detecting ticks in the environment through a standardized protocol. Active field dragging may capture some adventitious ticks (nonnative ticks introduced most likely by migratory birds), but adventitious ticks are unlikely to play a significant role particularly if the region sampled is suspected suitable for ticks based on other surveillance reports [39, 40]. Therefore, we defined presence points for *I*. *scapularis* as unique georeferenced locations where adult, nymph, or larvae of *I*. *scapularis* were found. We defined presence points for *B*. *burgdorferi* as unique georeferenced locations where at least one positive *I*. *scapularis* specimen was found.

To further ensure our presence points represent an unbiased sample of the species’ niche, we created a gridded Gaussian kernel density sampling bias file based on all 120 sites we surveyed for ticks between 2015 and 2018, following the approach described by Brown *et al*. [41]. This approach was selected because it allowed Maxent to sample pseudo-absences from background with the same distribution that gave rise to presence points while accounting for higher density of sampling in specific regions. Lastly, we also explored spatial autocorrelation in the presence point data for *I*. *scapularis*, and for *B*. *burgdorferi*. We detected highly spatially clustered presence points around the city of Ottawa and in other regions of eastern Ontario with a high number of sampling locations. To avoid pseudo-replication of data points and reduce clustering, we rarefied species occurrence points by creating circular buffer zones in 1 km increments around each site and randomly selecting one location in overlapping zones [41, 42]. We used nearest neighbour analysis and the z-score and associated p-value to select the buffer size that would ensure a random distribution was achieved while keeping the maximum number of data points (S3 Table) [41, 42].

To select ecological parameters for our Maxent models for *I*. *scapularis* and *B*. *burgdorferi*, we first evaluated the importance of the 41 variables we derived from earth observation data by grouping the variables into land cover and climate groups and running “full models” with default Maxent settings for each category. We ranked variables by their contribution to the model as measured by average loss in regularized training gain when each variable was omitted in turn and we included explanatory variables with greater than 1% decrease in gain in our main models [43]. We then used the ArcMap extension SDM Toolbox v.2.4 to derive a correlation matrix and we removed highly correlated variables (|Pearson’s r| ≥ 0.7) starting with the lowest contributing variables [41, 44]. For land cover, we also ensured that each variable (i.e. measured as proportion or distance) was only represented once in the final model to avoid duplicate contributions of the same variable. We analyzed land cover and climate variables separately because, while both contribute to tick establishment, climate varies much less at a fine resolution and would be underrepresented in the final model. S4 and S5 Tables show the selection of the explanatory variables for the *I*. *scapularis* and *B*. *burgdorferi* models, respectively.

Using the final set of land cover and climate variables with moderate to low correlation, we selected the types of variable transformations to use in our model. In machine learning, these transformations, or functions, of the original variables (i.e. linear, quadratic, product, hinge, etc.) are called features and can be used to fit highly complex models [25]. However, models with a larger number of features tend to overfit small training datasets and are more difficult to interpret [45]. For models with 30–40 training points, linear-quadratic-product (LQP) features typically produce the best performance while smaller datasets perform well with linear-quadratic (LQ) features when evaluated using area under the receiver operating characteristics curve (AUC) [45]. For our *I*. *scapularis* and *B*. *burgdorferi* models, we used LQP features and LQ features, respectively.

### Model calibration

To calibrate the models, we used the threshold-independent receiver operating characteristic (ROC) analysis and the threshold-dependent omission rate [46]. The area under the ROC function (AUC) quantifies the probability that the model correctly ranks a random presence locality higher than a random background locality [38]. Thus, AUC can be used to measure model performance (i.e. discriminatory ability) compared to random prediction. The omission rate requires a threshold to be selected in order to produce a binary prediction (i.e. suitable, not suitable) and is defined as the fraction of presence localities that fall into pixels not predicted as suitable [38]. We defined the threshold as the lowest prediction value for any pixel that holds a training presence point, which indicates the least suitable environmental conditions in which a presence can be found. We compared omission rates for our testing models with the theoretical predicted omission rate of zero for this threshold [46].

We used the 4-fold cross validation method described by Radosavljevic *et al*., 2014 to partition our data and use all presence points for training and testing [46]. We then generated models with different regularization values and measured AUC and omission rate to assess fit (S1 Fig). Regularization reduces model overfitting by ensuring empirical constraints are not fit too tightly and by removing features from the model and reducing model complexity [47]. We selected the best model with a regularization value that produced the lowest omission rate (i.e. closest to the predicted value of zero) and highest AUC based on the testing data (S1 Fig).

### Validation and variable contribution

**Fig 2:**
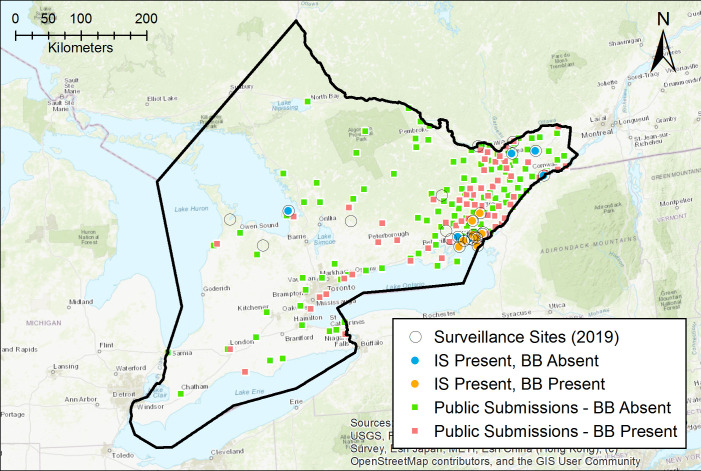
Model validation datasets: tick field sampling conducted in 2019 and publicly submitted ticks between 2015–2017. Open circles show sites sampled for ticks in 2019, blue circles indicate sites where *Ixodes scapularis* (IS) ticks were found, orange circles show sites where *Borrelia burgdorferi* (BB) was detected through molecular testing, green squares show a random subset of sites where the public encountered ticks negative for *B*. *burgdorferi* between 2015–2017, and pink squares show a random subset of sites where the public encountered ticks positive for *B*. *burgdorferi* between 2015–2017.

To validate the predictive ability of our final models for *I*. *scapularis* and *B*. *burgdorferi*, we used two independent datasets that were generated from contemporary tick surveillance in the province. First, we used active tick surveillance data from 2019 where 25 unique sites in southern and eastern Ontario were sampled for ticks via drag sampling over an area of 2000 m^2^ per site (Kulkarni et al., unpublished data) (Fig 2). *Ixodes scapularis* ticks were found at 14 of these sites and *B*. *burgdorferi* at 8 of these sites. Second, we used contemporary passive tick surveillance data from 2015–2017 from Public Health Ontario to generate a second dataset with locations in the province where individuals have encountered ticks (Fig 2). We retained georeferenced records with high level of spatial certainty on location of tick acquisition (e.g. specific address, park, trail) and excluded records with imprecise locations or locations outside the study area. After rarefying data to avoid pseudo-replication of data points, we had 106 unique locations where blacklegged ticks were encountered by the public and 63 locations with *B*. *burgdorferi*-infected specimens across our study area. We then used AUC to assess the discriminatory ability of our models.

Furthermore, we measured the variable contribution of our final models for *I*. *scapularis* and *B*. *burgdorferi* using three metrics. First, we used a jackknife procedure to assess the regularized training gain of models built using each variable individually. Second, we used a jackknife procedure to assess regularized training gain when each variable is excluded from the model in turn. Lastly, we used permutation importance obtained by randomly permuting the values of each variable on presence and background points and re-evaluating the model with these values and measuring the decrease in AUC. Independent response curves for each variable were also used to assess how each variable affects the predicted suitability of the two species.

## Results

### Ecological niche models for *Ixodes scapularis* and *Borrelia burgdorferi* in Ontario

Using field surveillance data, we identified 48 spatially independent locations where established populations of *I*. *scapularis* ticks were found; these were used as occurrence records for model development (Fig 1). The final niche model for *I*. *scapularis* included 12 environmental variables, used linear, quadratic, and product transformations of the environmental variables, imposed a regularization multiplier of 2 to avoid overfitting, and presented environmental suitability from 0 to 1 using the clog log transformation (Tables 1 and 2). The final *B*. *burgdorferi* model was developed using 30 spatially independent locations where ticks tested positive for the bacterium and included 12 environmental variables, used linear and quadratic transformations of the environmental variables, and a regularization multiplier of 1.5 (Tables 1 and 3).

**Table 1:** Maxent parameters and fit metrics of the best models based on 4-fold cross validation for *Ixodes scapularis* and *Borrelia burgdorferi*.

	*Ixodes scapularis* model	*Borrelia burgdorferi* model
**Parameter**		
Presence points, *n*	48	30
Variables, *n*	12	12
Features types	linear, quadratic, product	linear, quadratic
Regularization multiplier	2	1.5
Output	clog log	clog log
**4-fold cross validation**		
Mean AUC	0.925	0.963
Mean omission rate	0.0415	0.10275

**Table 2:** Measures of variable contribution for covariates in the *Ixodes scapularis* model.

Variable	Permutation importance	Gain without variable	Gain with only variable
Distance to coniferous forest	24.7	1.1137	0.1383
Distance to deciduous forest	22.6	1.0479	0.1309
Elevation	19.1	0.9809	0.4021
DD>0°C	13.2	1.0713	0.0097
Proportion of agriculture	8.7	1.0194	0.1564
Proportion of rural or undifferentiated land	4.7	1.0578	0.0065
Precipitation of warmest quarter	2.2	1.0926	0.0154
Precipitation of wettest quarter	1.9	1.1392	0.0246
Temperature seasonality	1.4	1.1363	0.0207
Distance to water	0.8	1.1184	0.0197
Proportion of infrastructure	0.5	1.0948	0.0681
Proportion of hedge rows	0.2	1.1394	0.0176
Full model regularized training gain: 1.1494			

**Table 3:** Measures of variable contribution for covariates in *Borrelia burgdorferi* model.

Variable	Permutation importance	Gain without variable	Gain with only variable
Proportion of agriculture	22.7	1.4827	0.2272
Proportion of water	18.7	1.5657	0.3319
Distance to mixed treed forest	17.4	1.6617	0.2433
Mean temperature of warmest quarter	14.5	1.6327	0.1245
Elevation	10.0	1.6289	0.4886
Precipitation of coldest quarter	6.3	1.6969	0.0917
Proportion of rural or undifferentiated land	4.9	1.6176	0.1144
Proportion of infrastructure	2.3	1.5856	0.1815
Precipitation of warmest quarter	1.7	1.6836	0.0267
Mean temperature of driest quarter	1.1	1.7207	0.0917
Proportion of coniferous forest	0.3	1.707	0.0592
Proportion of hedge rows	0.2	1.7112	0.0539
Full model regularized training gain: 1.7412			

**Fig 3:**
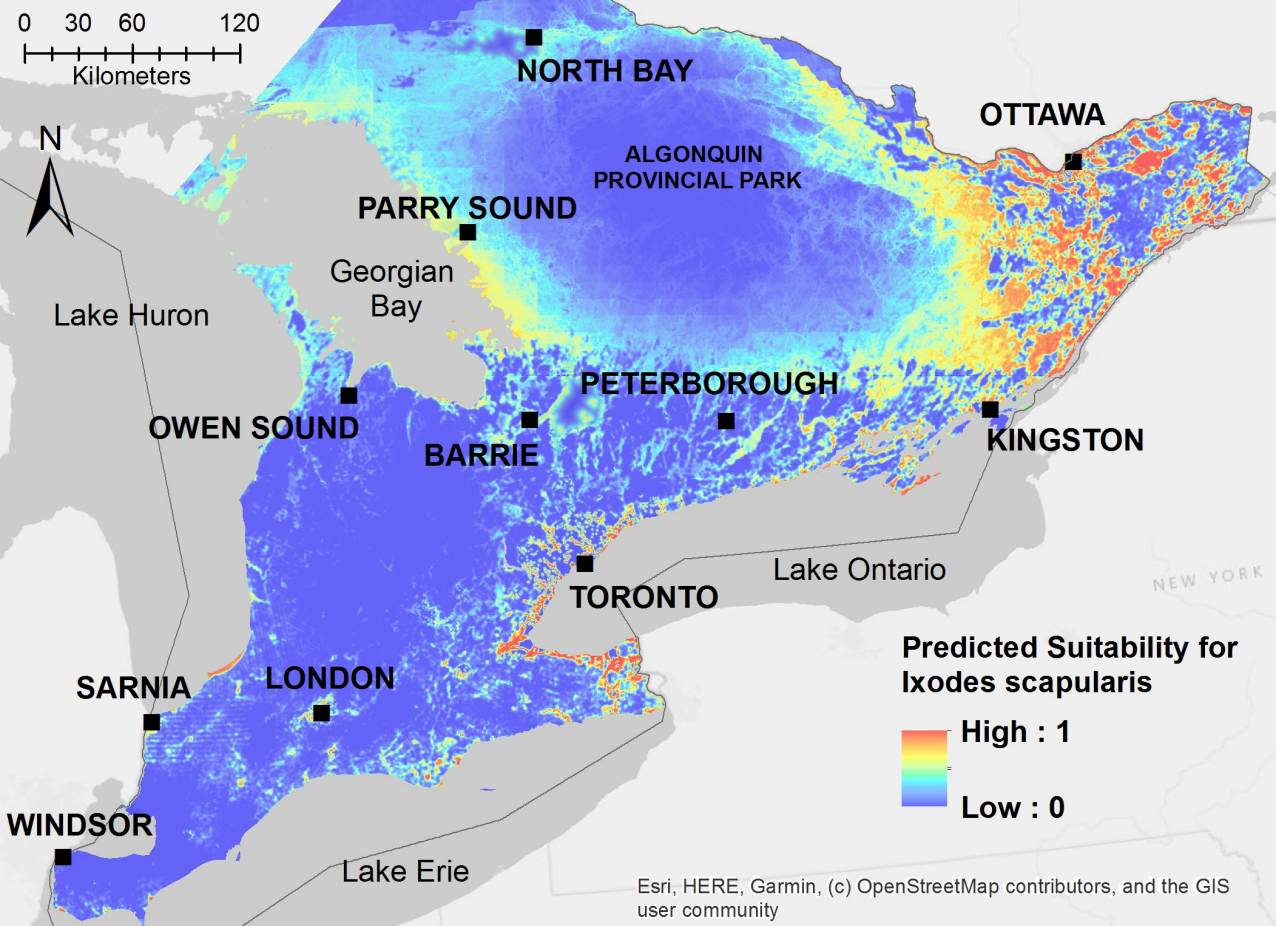
Ecological niche model for *Ixodes scapularis* derived from 48 spatially independent presence locations with established tick populations. Model output with a clog log transformation to show the predicted suitability for *I*. *scapularis* as a probability from 0–1.

**Fig 4:**
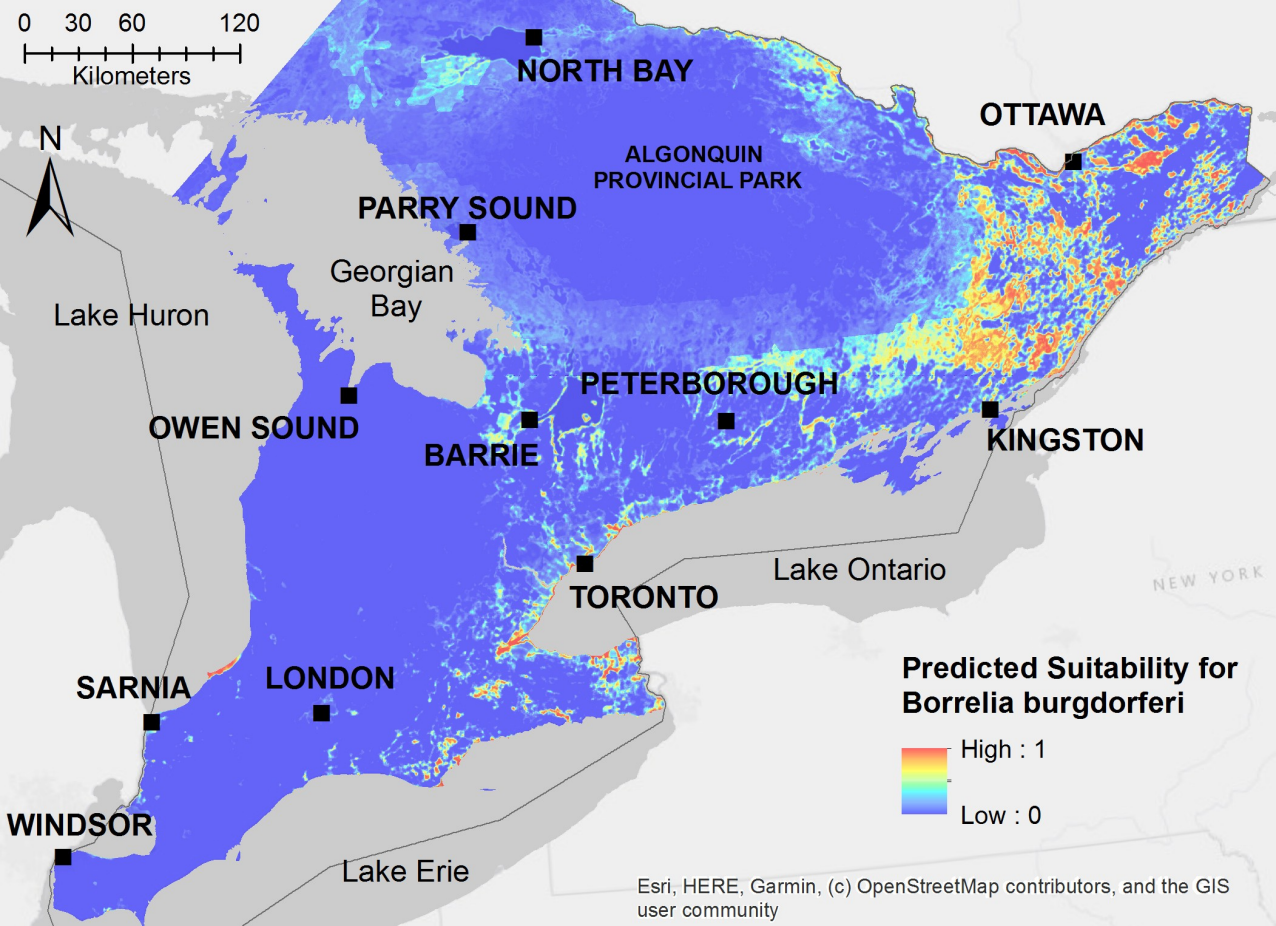
Ecological niche model for Borrelia burgdorferi derived from 30 spatially independent presence locations with established tick populations and positive specimens for the bacterium. Model output with a clog log transformation to show the predicted suitability for *B*. *burgdorferi* as a probability from 0–1.

Our model for *I*. *scapularis* predicts the highest suitability for this species in eastern Ontario, particularly the regions between the cities of Kingston and Ottawa (Fig 3). Localized high suitability for *I*. *scapularis* was also predicted along the shores of Lake Ontario and to a lesser extent in areas around Lake Erie, Lake Huron, and the Georgian Bay (Fig 3). *Ixodes scapularis* habitat suitability was lowest in the agricultural region of southern Ontario and in the central region of Algonquin Provincial Park (Fig 3). Our model for *B*. *burgdorferi* predicted that the pathogen’s range is narrower than that of the blacklegged tick and almost entirely constrained to eastern Ontario, with a few localized areas around the shores of Lake Ontario and Lake Erie (Fig 4).

Based on model validation using occurrence points from active tick surveillance in 2019, our niche models demonstrated good discrimination of positive and background sites for both *I*. *scapularis* (AUC = 0.898) and *B*. *burgdorferi* (AUC = 0.958) (Table 4). Additionally, we used passive tick surveillance to generate another independent dataset consisting of ticks voluntarily submitted by the public to local health units. Based on model validation using the dataset from passive tick surveillance in 2015–2017, our niche models demonstrated good discrimination of positive and background sites for *I*. *scapularis* (AUC = 0.727) and *B*. *burgdorferi* (AUC = 0.863) (Table 4), although with slightly lower AUC values as would be expected given the lower precision of tick occurrence locations and possible inclusion of locations where individuals encountered adventitious ticks.

**Table 4:** Validation of *Ixodes scapularis* and *Borrelia burgdorferi* models with two independent datasets derived from active and passive surveillance activities in Ontario, Canada.

	*Ixodes scapularis* model	*Borrelia burgdorferi* model
**Training AUC**	0.950	0.981
**Test AUC**		
Active surveillance 2019	0.898	0.958
Passive surveillance 2010–2017	0.727	0.863

### Environmental variables contributing to tick habitat suitability and establishment of *Borrelia burgdorferi*

**Fig 5:**
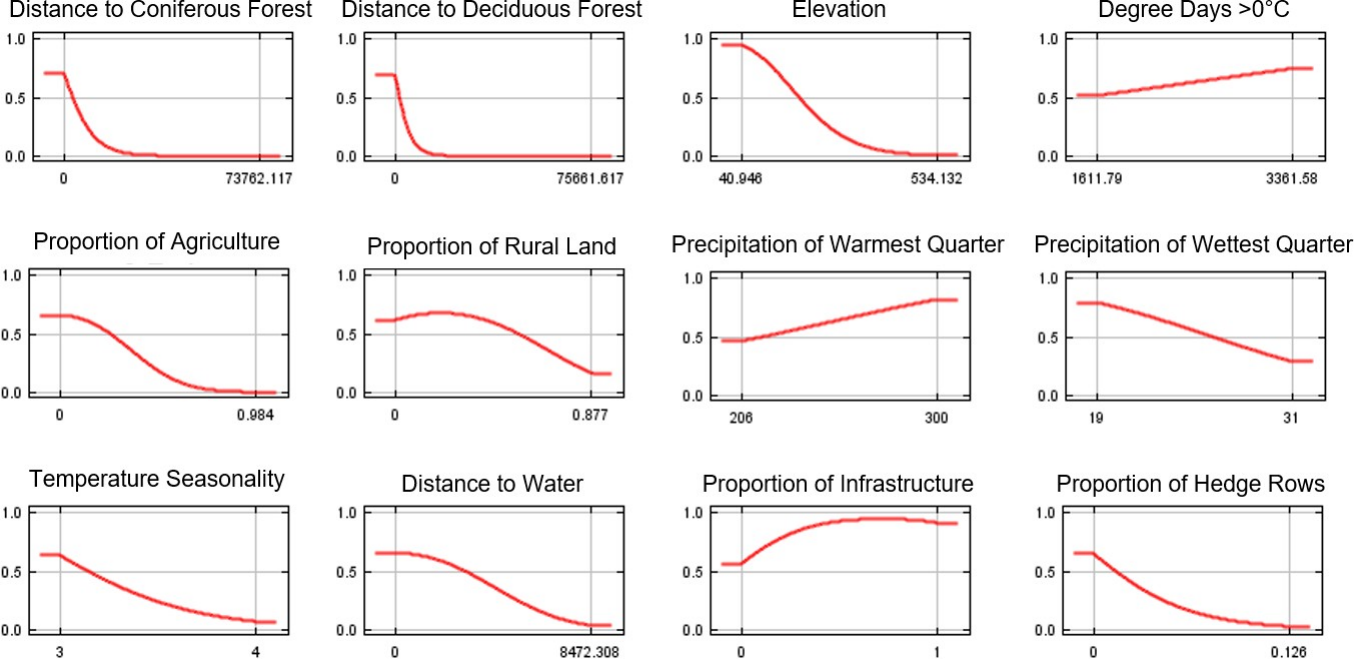
Independent response curves showing the dependence of predicted suitability for *Ixodes scapularis* on the variables modelled in turn.

We used two jackknife procedures to resample our data and measure the regularized training gain when variables were omitted in turn from the models or considered in isolation in the models. For our *I*. *scapularis* model, the variables whose model gain decreased the most when omitted were, in turn, elevation, proportion of agricultural land, and distance to deciduous forest (Table 2). Similarly, these variables also increased the model gain the most when considered in isolation, indicating they are the most informative variables in the model containing information not found in the other variables (Table 2). The variables with the highest permutation importance that contribute the most to model fit include distance to coniferous forest, distance to deciduous forest, elevation, and DD>0°C (Table 2). Based on independent response curves, habitat suitability for blacklegged ticks increases with increasing DD>0°C and decreases with larger distances to coniferous and deciduous forests and with higher elevation (Fig 5).

**Fig 6:**
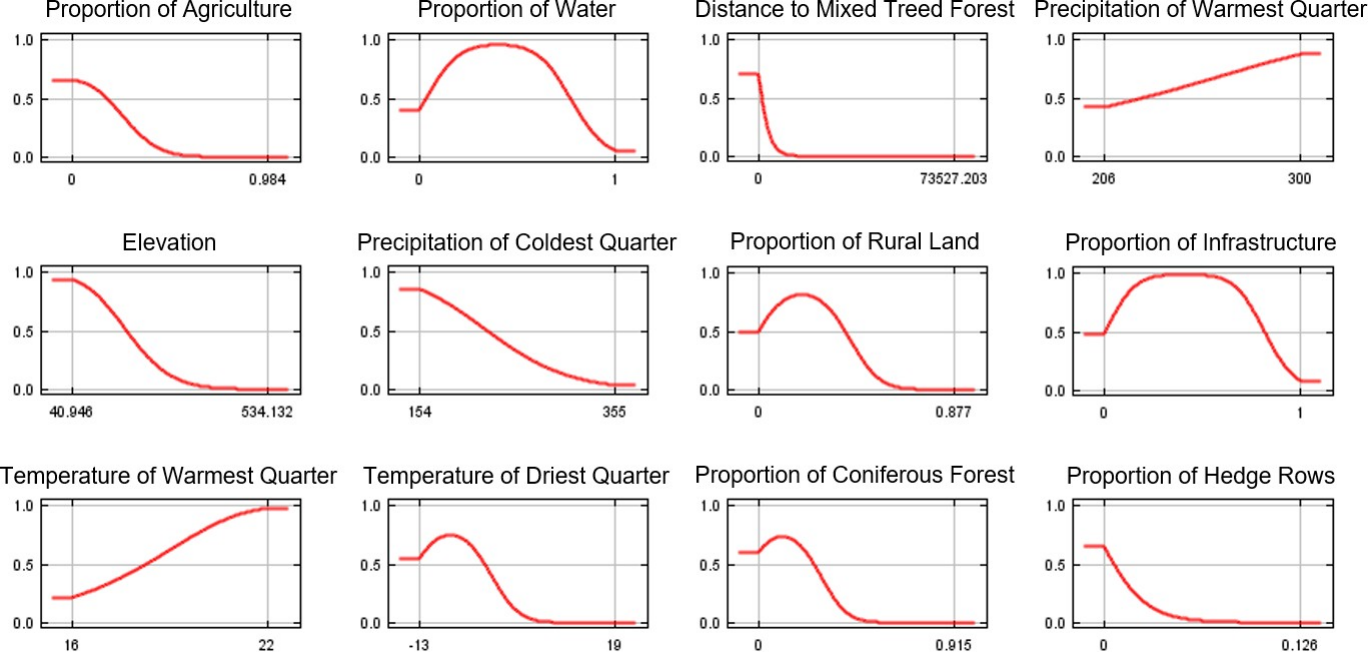
Independent response curves showing the dependence of predicted suitability for *Borrelia burgdorferi* on the variables modelled in turn.

Similarly, for the *B*. *burgdorferi* model, proportion of agricultural land, elevation, and distance to mixed treed forest were among the most informative variables (Table 3). However, proportion of water and proportion of infrastructure were also highly informative variables to the predicted suitability of the pathogen in Ontario (Table 3). Environmental suitability for this pathogen increased with higher mean spring/summer temperature and decreased with higher elevation, higher proportion of agricultural land, and larger distance to mixed treed forest based on independent variable response curves (Fig 6). Increasing proportions of infrastructure and surrounding area composed of open water also increased environmental suitability for *B*. *burgdorferi* but decreased suitability when the proportions reached a greater threshold, which likely explains why shorelines along Lake Ontario with major population centers are predicted to be highly suitable for the establishment of ticks and tick-borne pathogens.

## Discussion

In this study, we developed environmental risk maps for Lyme disease in southern and eastern Ontario by modelling the vector’s predicted habitat suitability. We also developed a second model for the Lyme disease-causing bacterium, *B*. *burgdorferi*, to identify the suitability of this region to sustain the transmission cycle of this pathogen. The *I*. *scapularis* and *B*. *burgdorferi* risk maps were developed using a maximum entropy modelling approach based on presence data derived from active field sampling at 120 sites across the province and environmental variables derived from high-resolution earth observation data.

Our *I*. *scapularis* model predicted high habitat suitability for blacklegged ticks throughout eastern Ontario and along the shorelines of Lake Ontario, where major population centers are located. This region is encompassed within the Great Lakes-St. Lawrence forest region, which is dominated by hardwood forests featuring maple, oak, birch, and pine and is home to a variety of wildlife including white-tailed deer, moose, small mammals, and migratory birds [48, 49]. The predicted habitat suitability for *I*. *scapularis* in our model is consistent with other studies that have examined the recent distribution and expansion of *I*. *scapularis* in Ontario [14, 30, 50]. However, our model also detected moderate habitat suitability for *I*. *scapularis* along the Georgian Bay and regions bordering Algonquin Provincial Park. Recent studies have shown that range expansion of *I*. *scapularis* populations in Ontario was limited to a northward movement of ticks in the eastern part of the province and that the odds of detecting *I*. *scapularis* decreased at sites located west of major endemic regions [21, 30]. The limited horizontal (westward) expansion of ticks in central and eastern Ontario may be explained by the large amount of agricultural land between suitable woodland habitats as well as by a larger distance from endemic sites in the northeastern United States, from which migratory birds are transporting ticks along the Atlantic and Mississippi flyways [21, 51]. Our model complements these findings by showing the predicted distribution of *I*. *scapularis* in Ontario, which may extend beyond the vector’s current presence, as a result of Maxent’s projection into geographic areas not directly sampled for ticks [38, 52].

It is interesting that Algonquin Park and regions of northern Ontario have a lower predicted probability of blacklegged tick occurrence. This is probably a result of higher elevation, which has been shown to be a strong predictor of tick occurrence globally [21, 22, 53]. However, landscape structure and connectivity as well as host abundance and diversity may also play a role in the establishment of ticks in forest habitats. It had been shown that *I*. *scapularis* (and its western counterpart *I*. *pacificus*) is predominantly found in woodlands with high canopy cover compared to open shrublands, yet, on the other hand forest fragmentation contributes to higher prevalence of *B*. *burgdorferi* along forest edges and urban forests [22, 54, 55]. It is currently unclear how landscape structure affects the colonization ability of *I*. *scapularis* and why regions like Algonquin Park, with very dense mixed forests, show a lower suitability for ticks. It is also possible that landscape barriers, such as a larger distance to open water and migratory bird flyways, may affect the movement of ticks to this region. Therefore, the apparent low suitability for ticks in Algonquin Park may be a result of a colonization lag that prevented us from findings ticks in this region through active surveillance. There is some evidence from our passive surveillance validation dataset that *I*. *scapularis* ticks have been reported in Algonquin Park by the public; however, these ticks represent a very low proportion compared to those found in eastern Ontario. It is also more difficult to interpret the accuracy of passive tick surveillance data due to possible recall bias. Overall, we believe a that Algonquin Park and the surrounding region represents a lower risk of exposure to ticks and Lyme disease compared to areas in eastern Ontario with high tick densities and a higher prevalence of *B*. *burgdorferi*, though additional research is needed to explore newly emerging areas and dense forests in Ontario.

We found that distance to coniferous forest, distance to deciduous forest, elevation, and DD>0°C were the variables that contributed most to our *I*. *scapularis* niche model. Our results are supported by other findings showing a positive association between tick abundance and forest/woodland habitat, warming temperatures, and lower elevations [6, 15, 21, 22, 56]. In our model, total DD>0°C was found to be an important factor for tick habitat suitability, but this variable was outweighed in importance by land cover features. In recent studies, Clow *et al*. found that the log odds of *I*. *scapularis* presence was correlated with increasing DD>0°C but that ecological factors such as forest type were not significant for *I*. *scapularis* colonization of new sites [21, 30]. A key difference is that our model used spatial measures of land cover derived from earth observation data rather than site-level descriptors; furthermore, it was calibrated on a different definition of tick presence and more recent years to identify environments capable of sustaining stable tick populations, where humans are most likely to encounter ticks. Cumulatively, these results indicate that climatic variables, which are more uniform over large geographic regions, are important in driving tick expansion and colonization of new areas, whereas ecological variables, which have potentially high variability at a local scale, play an important role in sustaining tick populations after initial site colonization.

Recent studies have also focused on microclimate or microhabitat to identify *I*. *scapularis* distributions at a local scale, and have found significant associations between nymphal and adult tick densities with forest type, forest understory, dominant tree type, depth of litter layer, distance from trails, type of trails, and distance to roads, which support our findings for the dominance of forests in *I*. *scapularis* habitat suitability [15, 21, 22, 57]. A local ecological niche model for *I*. *scapularis* in the city of Ottawa also found that distance to forests and treed land were among the strongest variables predicting the distribution of blacklegged ticks [24]. Deciduous forests have been shown to be most favourable for *I*. *scapularis* establishment in other studies in North America, while coniferous forests were least favourable for ticks [58, 59]. However, in our model distance to both types of forest were found to be important for *I*. *scapularis* habitat, which may reflect the importance of cedar and maple forests for tick density [22]. This is also likely due to the behaviour of white-tailed deer, the main reproductive host for *I*. *scapularis*, which frequent forest edges that are dominated by coniferous trees such as white cedar, eastern hemlock, and white pines [20, 60].

Interestingly, in our model the predicted distribution of *B*. *burgdorferi* was heavily concentrated in eastern Ontario and limited to the high-probability *I*. *scapularis* regions. It is currently unclear if this reflects the lag-phase between tick establishment and infection with *B*. *burgdorferi* or whether local scale factors promote the establishment of *B*. *burgdorferi* in some regions of Ontario versus others. There are three hypotheses for the emergence of *B*. *burgdorferi*: tick first, pathogen first, or dual infection [4, 30]. In Canada, studies from passive and active surveillance support the tick-first hypothesis where ticks are brought into the region by migratory birds, followed, in eastern regions, by an estimated five-year lag between tick establishment and transmission of *B*. *burgdorferi* [28, 51, 61]. Our results support this hypothesis since the distribution of *B*. *burgdorferi* mirror that of *I*. *scapularis* but is more constrained in eastern Ontario. However, we cannot rule out the possibility that other factors, such as abundance of white-footed mice, habitat fragmentation, or other underlying ecological and biological factors favour the establishment of *B*. *burgdorferi* in specific regions independently of *I*. *scapularis*.

Based on our results, elevation, proportion of agriculture, distance to mixed forest, proportion of water, and proportion of infrastructure are the most important and informative variables predictive of *B*. *burgdorferi* distribution. While there are similarities in the types of variables that contribute to both models such as distance to mixed forest, elevation, and proportion of agriculture, the distribution of *B*. *burgdorferi* is more dependent on the proportion of infrastructure and water than that of *I*. *scapularis*. In a recent study of landscape determinants for blacklegged ticks in the Ottawa-Gatineau region, Talbot *et al*.,2019 also found that distance to roads was a significant predictor of *B*. *burgdorferi* infection prevalence [22]. The importance of infrastructure and urban development in this model may be explained by local adaptations to urbanization of the white-footed mouse, which is the main reservoir for *B*. *burgdorferi*, or the role of other small mammals as competent reservoirs for *B*. *burgdorferi* in regions of the province [62–64]. Additionally, the importance of water in the *B*. *burgdorferi* model may represent possible habitat requirements of key reservoir species or hosts for *I*. *scapularis* as well as entry points of infected ticks via migratory birds [24, 51, 65].

Our *I*. *scapularis* and *B*. *burgdorferi* models showed good discrimination of positive and negative sites when validated against two independent datasets, indicating that the predicted distributions of *I*. *scapularis* and *B*. *burgdorferi* are supported by the currently available data. Our results are also consistent with human Lyme disease incidence rates in the province, with eastern Ontario health units reporting the highest incidence rates per 100,000 population [9, 14]. This further demonstrates that areas of highest environmental risk are strongly correlated with areas of highest human Lyme disease incidence, although this is not the case in all parts of the world [66]. Thus, assessing environmental risk is important for informing tick and human disease surveillance, especially in areas that are predicted as suitable by the model but have limited ongoing surveillance, and to target disease prevention and control to populations living in the highest-risk regions.

Our study has several important strengths. First, we calibrated our models based on multi-year active tick surveillance data with a high degree of spatial accuracy. We modelled habitat suitability based on locations with detected tick occurrences, defined by presence of ticks in the environment and public submissions from nearby regions, to identify geographic areas where humans and animals are most likely to encounter ticks and tick-borne pathogens. Second, we used high resolution earth observation data from which we derived a large number of environmental variables on climate, elevation, and land cover that might affect the ecology of these species and we used a machine learning approach to model the predicted distribution of the species. Lastly, we rigorously validated our models with two independent datasets: active tick surveillance at new sites (i.e. not used for model calibration) and contemporary passive tick surveillance, representing sites where the public has encountered ticks.

Our study also has several limitations including the unavailability of certain variables such as forest fragmentation and other microhabitat features that are relevant on a local scale and may help explain some observed difference between the ecological requirements of *I*. *scapularis* versus *B*. *burgdorferi*. Additionally, we were unable to account for all sampling biases that may arise from operator experience, daily conditions during dragging, and protocol variations as well as other factors that affect tick presence and/or abundance such as the distribution of tick hosts (e.g. densities of white-tailed deer and white-footed mice), barriers to host dispersal, human habitat modifications, and seasonal variations [67, 68]. SDMs have also been criticized for generating more conservative estimates of a species’ distribution because they are empirical models that rely on occurrence points for calibration and, therefore, cannot predict the full extent of a species’ niche [23, 38, 69]. Furthermore, SDMs like ours should also be interpreted with caution because they project inferences based on associations between tick occurrence and environmental variables into target geographic areas, and do not reflect tick abundance or the actual distribution of ticks [67]. Our models simply predict the potential distribution of ticks and pathogens in southeastern Ontario, and thereby provide one possible measure of risk.

## Supporting information

S1 Fig Four-fold cross validation showing the average area under the receiver operating characteristic curve (AUC) (a) and omission rate (b) for the minimum training presence threshold. The best model for *Ixodes scapularis* and *Borrelia burgdorferi* were selected to minimize omission rate and maximize AUC.
(TIF)

S1 Table Compilation raster for land cover. Mosaic of the Southern Ontario Land Resource Information System version 3.0 (SOLRISv3.0) captured between 2000–2015 and the Provincial Land Cover Database 2000 (PLCD2000) UTM zones 17 and 18 captured between 1999–2002.
(DOCX)

S2 Table Raster processing and derivation of land cover, elevation and climate variables. (DOCX)

S3 Table Average nearest neighbour analysis to select the buffer size used to rarefy the *Ixodes scapularis* and *Borrelia burgdorferi* presence points used to develop the niche model. (DOCX)

S4 Table Selection of environmental variables for the *Ixodes scapularis* model. (DOCX)

S5 Table Selection of environmental variables for the *Borrelia burgdorferi* model. (DOCX)

S1 Data (XLS)
