# Supplementary figures and images for: Species distribution models for the eastern blacklegged tick, Ixodes scapularis, and the Lyme disease pathogen, Borrelia burgdorferi, in Ontario, Canada
Source: PLoS One. 2020 Sep 11;15(9):e0238126. doi: 10.1371/journal.pone.0238126 (PMC7485816; doi:10.1371/journal.pone.0238126)

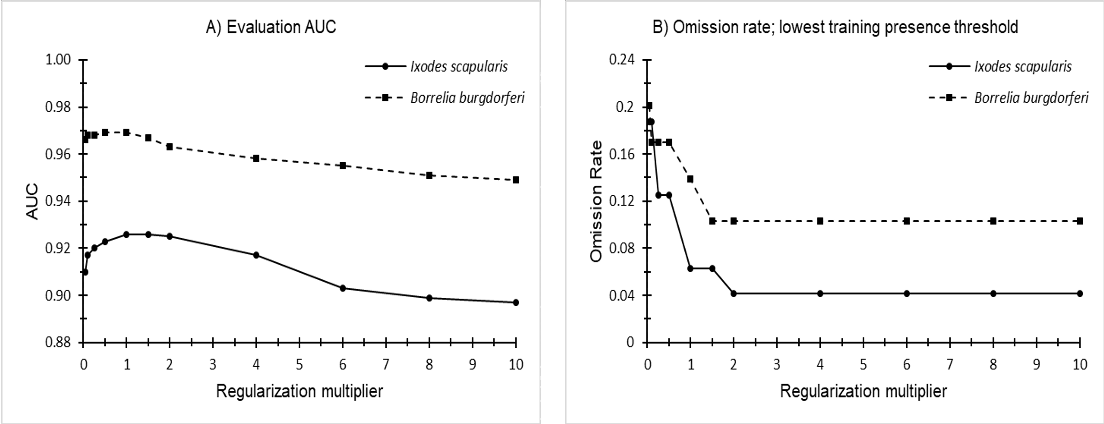

Supplement: S1 Fig — Four-fold cross validation showing the average area under the receiver operating characteristic curve (AUC) (a) and omission rate (b) for the minimum training presence threshold. The best model for Ixodes scapularis and Borrelia burgdorferi were selected to minimize omission rate and maximize AUC. (TIF) [file pone.0238126.s001.tif]
