# Supplementary material for: Species distribution models for the eastern blacklegged tick, Ixodes scapularis, and the Lyme disease pathogen, Borrelia burgdorferi, in Ontario, Canada
Source: PLoS One. 2020 Sep 11;15(9):e0238126. doi: 10.1371/journal.pone.0238126 (PMC7485816; doi:10.1371/journal.pone.0238126)
Supplement: S1 Table — Mosaic of the Southern Ontario Land Resource Information System version 3.0 (SOLRISv3.0) captured between 2000–2015 and the Provincial Land Cover Database 2000 (PLCD2000) UTM zones 17 and 18 captured between 1999–2002. (DOCX) [file pone.0238126.s002.docx]

**S1 Table.** **Compilation raster for land cover.** Mosaic of the Southern Ontario Land Resource Information System version 3.0 (SOLRISv3.0) captured between 2000-2015 and the Provincial Land Cover Database 2000 (PLCD2000) UTM zones 17 and 18 captured between 1999-2002.

| **Unit Name** | **Source Unit Name** | **Source Unit Description** |
| --- | --- | --- |
| Water | PLC2000: *Water – deep clear* | *Water – deep clear:* Deep or clear waterbodies. |
|  | SOLRISv3.0: *Open water* | *Open water:* Water depth > 2 meters. Lake trophic status. No macrophyte vegetation, trees or shrub cover. |
| Agricultural land | PLC2000: *Cropland* | *Cropland:* Areas of row crops and fallow fields. |
|  | SOLRISv3.0: *Tilled* | *Tilled:* Agricultural fields managed as continuous annual row crops inferred from 3 observed sequential time periods over a 10-year time period. There can be as many as 2 time periods where fields are rotated with perennial crops. (e.g., hay, improved pasture). |
| Undifferentiated of rural land | PLC2000: *Pasture* | *Pasture:* Open grassland with sparse shrubs in rural land. |
|  | SOLRISv3.0: *Undifferentiated* | *Undifferentiated:* Includes some agricultural features not included in tilled (i.e. orchards, vineyards, perennial crops and idle land > 10 years – out of agricultural production) as well as urban brown fields, hydro and transportation right-of- ways, upland thicket and openings within forests. |
| Deciduous forest | PLC2000: *Deciduous forest* | *Deciduous forest:* Largely continuous forest canopy composed primarily of deciduous species. |
|  | SOLRISv3.0: *Deciduous forest* | *Deciduous forest:* Tree cover > 60%. Upland deciduous tree species > 75% canopy cover > 2m in height. |
| Coniferous forest | PLC2000: *Coniferous forest* | *Coniferous forest:* Largely continuous forest canopy composed primarily of coniferous species |
|  | SOLRISv3.0: *Coniferous forest* | *Coniferous forest:* Tree cover > 60%. Upland coniferous tree species > 75% canopy cover > 2m in height. |
| Mixed forest | PLC2000: *Mixed forest* | *Mixed forest:* Largely continuous forest canopy composed of both deciduous and coniferous species. In more northerly areas, a greater component of coniferous species can be expected; in more southerly areas, a greater component of deciduous species can be expected. |
|  | SOLRISv3.0: *Mixed forest*, *Forest* | *Mixed forest:* Tree cover > 60%. Upland coniferous tree species > 25%and deciduous tree species > 25% of canopy cover > 2m in height.  *Forest:* Tree cover > 60%. Upland tree species > 75% canopy cover > 2m in height. Attribute for forest type could not be derived spectrally from Landsat automated analysis due to size of feature. |
| Infrastructure | PLC2000: *Settlement / Infrastructure* | *Settlement / Infrastructure:* Clearings for human settlement and economic activity; major transportation routes. |
|  | SOLRISv3.0: *Transportation*, *Built-up area – pervious, Built-up area – impervious* | *Transportation:* Highways, roads.  *Built-up area – pervious:* Urban recreation areas. (i.e., golf courses, playing fields)  *Built-up area – impervious:* Residential, industrial, commercial, and civic areas. |
| Hedge rows | PLC2000: -- | -- |
|  | SOLRISv3.0: *Hedge rows* | *Hedge rows*: Tree cover > 60%, (trees > 2m height), linear arrangement, minimum 10 meters width, maximum 30 meters width. |
| Marsh | PLC2000: *Intertidal marsh, Supertidal marsh, Inland marsh* | *Intertidal marsh:* Coastal marshes of the Hudson Bay-James Bay Lowlands lying between the coastal mudflats and the supertidal zone.  *Supertidal marsh:* Coastal marshes of the Hudson Bay-James Bay Lowlands lying inland of both the coastal mudflats and intertidal marshes, and subject to only exceptionally high tides.  *Inland marsh:* Lakeshore and inland marshes of Southern Ontario. |
|  | SOLRISv3.0: *Marsh* | *Marsh:* Open and shrub communities. Water table seasonally or permanently at, near, or above substrate surface – tree and shrub cover <=25%. Dominated by emergent hydrophytic macrophytes. |
| Sparse Treed | PLC2000: *Sparse forest* | *Sparse forest:* A patchy or sparse forest canopy composed of coniferous or deciduous species or a combination of the two. |
|  | SOLRISv3.0: *Sparse treed:* | *Sparse treed:* 60% < tree cover < 10%. Confined to the Canadian Shield in ecoregion 5E. Tree communities often situated on non-calcareous bedrock features, rapidly draining soils, or raised mineral soils. |
