# Supplementary material for: Species distribution models for the eastern blacklegged tick, Ixodes scapularis, and the Lyme disease pathogen, Borrelia burgdorferi, in Ontario, Canada
Source: PLoS One. 2020 Sep 11;15(9):e0238126. doi: 10.1371/journal.pone.0238126 (PMC7485816; doi:10.1371/journal.pone.0238126)
Supplement: S2 Table — (DOCX) [file pone.0238126.s003.docx]

**S2 Table.** **Raster processing and derivation of land cover, elevation and climate variables.**

| **#** | **Variable** | **Processing** |
| --- | --- | --- |
|  | *Land Cover* |  |
| 1 | Distance to agricultural land | Calculated distance from each grid cell to a tilled agricultural cell |
| 2 | Proportion of agricultural land | Calculated proportion of tilled agricultural cells within a 1000 m buffer |
| 3 | Distance to coniferous forest | Calculated distance from each grid cell to a coniferous forest cell |
| 4 | Proportion of coniferous forest | Calculated proportion of coniferous forest cells within a 1000 m buffer |
| 5 | Distance to deciduous forest | Calculated distance from each grid cell to a deciduous forest cell |
| 6 | Proportion of deciduous forest | Calculated proportion of deciduous forest cells within a 1000 m buffer |
| 7 | Distance to hedge rows | Calculated distance from each grid cell to a hedge row cell |
| 8 | Proportion of hedge rows | Calculated proportion of hedge row cells within a 1000 m buffer |
| 9 | Distance to infrastructure | Calculated distance from each grid cell to an infrastructure cell |
| 10 | Proportion of infrastructure | Calculated proportion of infrastructure cells within a 1000 m buffer |
| 11 | Distance to marsh | Calculated distance from each grid cell to a marsh cell |
| 12 | Proportion of marsh | Calculated proportion of marsh cells within a 1000 m buffer |
| 13 | Distance to mixed forest | Calculated distance from each grid cell to a mixed forest cell |
| 14 | Proportion of mixed forest | Calculated proportion of mixed forest cells within a 1000 m buffer |
| 15 | Distance to rural or undifferentiated land | Calculated distance from each grid cell to a rural or undifferentiated land cell |
| 16 | Proportion of rural or undifferentiated land | Calculated proportion of rural or undifferentiated cells within a 1000 m buffer |
| 17 | Distance to sparse treed | Calculated distance from each grid cell to a sparse treed cell |
| 18 | Proportion of sparse treed | Calculated proportion of sparse treed cells within a 1000 m buffer |
| 19 | Distance to water | Calculated distance from each grid cell to a water cell |
| 20 | Proportion of water | Calculated proportion of water cells within a 1000 m buffer |
|  | *Climate* |  |
| 21 | Bio1: Annual mean temperature | Divided values by 10 and resampled raster at 100 meters resolution |
| 22 | Bio2: Mean diurnal range | Divided values by 10 and resampled raster at 100 meters resolution |
| 23 | Bio3: Isothermality | Divided values by 10 and resampled raster at 100 meters resolution |
| 24 | Bio4: Temperature seasonality | Divided raster values by 100 and resampled raster at 100 meters resolution |
| 25 | Bio5: Maximum temperature of warmest period | Divided values by 10 and resampled raster at 100 meters resolution |
| 26 | Bio6: Minimum temperature of coldest period | Divided values by 10 and resampled raster at 100 meters resolution |
| 27 | Bio7: Temperature annual range | Divided values by 10 and resampled raster at 100 meters resolution |
| 28 | Bio8: Mean temperature of wettest quarter | Divided values by 10 and resampled raster at 100 meters resolution |
| 29 | Bio9: Mean temperature of driest quarter | Divided values by 10 and resampled raster at 100 meters resolution |
| 30 | Bio10: Mean temperature of warmest quarter | Divided values by 10 and resampled raster at 100 meters resolution |
| 31 | Bio11: Mean temperature of coldest quarter | Divided values by 10 and resampled raster at 100 meters resolution |
| 32 | Bio12: Annual precipitation | Resampled raster at 100 meters resolution |
| 33 | Bio13: Precipitation of wettest month | Resampled raster at 100 meters resolution |
| 34 | Bio14: Precipitation of driest month | Resampled raster at 100 meters resolution |
| 35 | Bio15: Precipitation seasonality | Resampled raster at 100 meters resolution |
| 36 | Bio16: Precipitation of wettest quarter | Resampled raster at 100 meters resolution |
| 37 | Bio17: Precipitation of driest quarter | Resampled raster at 100 meters resolution |
| 38 | Bio18: Precipitation of warmest quarter | Resampled raster at 100 meters resolution |
| 39 | Bio19: Precipitation of coldest quarter | Resampled raster at 100 meters resolution |
| 40 | Bio20: Degree days ≥ 0°C | Resampled raster at 100 meters resolution |
|  | *Other* |  |
| 41 | Elevation | Resampled raster at 100 meters resolution |
