# Supplementary material for: Species distribution models for the eastern blacklegged tick, Ixodes scapularis, and the Lyme disease pathogen, Borrelia burgdorferi, in Ontario, Canada
Source: PLoS One. 2020 Sep 11;15(9):e0238126. doi: 10.1371/journal.pone.0238126 (PMC7485816; doi:10.1371/journal.pone.0238126)
Supplement: S3 Table — (DOCX) [file pone.0238126.s004.docx]

**S3 Table.** **Average nearest neighbour analysis to select the buffer size used to rarefy the *Ixodes scapularis* and *Borrelia burgdorferi* presence points used to develop the niche model.**

| ***Ixodes scapularis* model** | | | | |
| --- | --- | --- | --- | --- |
| **Buffer size** | **Number of points** | **z-score** | **p-value** | **Distribution** |
| - | 52 | -3.075624 | 0.002101 | clustered |
| 1 km | 51 | -2.677671 | 0.007414 | clustered |
| 2 km | 50 | -2.357250 | 0.018411 | clustered |
| 3 km | 48 | -1.352654 | 0.176166 | random |
| 4 km | 46 | -0.895225 | 0.370667 | random |
| 5 km | 44 | -0.705652 | 0.480404 | random |
|  |  |  |  |  |
| ***Borrelia burgdorferi* model** | | | | |
| **Buffer Size** | **Number of Points** | **z-score** | **p-value** | **Distribution** |
| - | 33 | -3.476831 | 0.000507 | clustered |
| 1 km | 33 | -3.476831 | 0.000507 | clustered |
| 2 km | 32 | -3.056015 | 0.002243 | clustered |
| 3 km | 30 | -1.512202 | 0.130482 | random |
| 4 km | 29 | -1.367204 | 0.171561 | random |
| 5 km | 27 | -0.976983 | 0.328578 | random |
