# Supplementary material for: Species distribution models for the eastern blacklegged tick, Ixodes scapularis, and the Lyme disease pathogen, Borrelia burgdorferi, in Ontario, Canada
Source: PLoS One. 2020 Sep 11;15(9):e0238126. doi: 10.1371/journal.pone.0238126 (PMC7485816; doi:10.1371/journal.pone.0238126)
Supplement: S4 Table — (DOCX) [file pone.0238126.s005.docx]

**S4 Table.** **Selection of environmental variables for the *Ixodes scapularis* model.**

| ***Ixodes scapularis* – Land cover variable selection** | | | | | |
| --- | --- | --- | --- | --- | --- |
|  | **Gain** | **% Decrease in Gain** | **Rank** | **Included in Model** | **Reason(s) Omitted** |
| **Full Model** | **1.9288** |  |  |  |  |
| Infra_Prop | 1.8326 | 4.99 | 1 | Yes |  |
| Rural_Prop | 1.8518 | 3.99 | 2 | Yes |  |
| Elevation | 1.8600 | 3.57 | 3 | Yes |  |
| Water_Dist | 1.8808 | 2.49 | 4 | Yes |  |
| Decid_Dist | 1.8840 | 2.32 | 5 | Yes |  |
| Conif_Dist | 1.8848 | 2.28 | 6 | Yes |  |
| Infra_Dist | 1.8869 | 2.17 | - | No | Variable already in model + Strong correlation |
| Hedge_Prop | 1.8900 | 2.01 | 7 | Yes |  |
| Agri_Prop | 1.9006 | 1.46 | 8 | Yes |  |
| Water_Prop | 1.9011 | 1.44 | - | No | Variable already in model |
| Conif_Prop | 1.9058 | 1.19 | - | No | Variable already in model |
| Decid_Prop | 1.9112 | 0.91 | - | No | Limited contribution + Variable already in model |
| Agri_Dist | 1.9178 | 0.57 | - | No | Limited contribution + Variable already in model |
| Sparse_Dist | 1.9185 | 0.53 | - | No | Limited contribution |
| Rural_Dist | 1.9186 | 0.53 | - | No | Limited contribution + Strong correlation |
| Marsh_Prop | 1.9191 | 0.50 | - | No | Limited contribution + Strong correlation |
| Mixed_Prop | 1.9211 | 0.40 | - | No | Limited contribution |
| Mixed_Dist | 1.9245 | 0.22 | - | No | Limited contribution |
| Marsh_Dist | 1.9289 | -0.01 | - | No | Limited contribution |
| Sparse_Prop | 1.9289 | -0.01 | - | No | Limited contribution |
| Hedge_Dist | 1.9294 | -0.03 | - | No | Limited contribution |
|  | | | | | |
| ***Ixodes scapularis* – Climate variable selection** | | | | | |
|  | **Gain** | **% Decrease in Gain** | **Rank** | **Included in Model** | **Reason(s) Omitted** |
| **Full Model** | **0.7354** |  |  |  |  |
| DD>0C | 0.6829 | 7.14 | 1 | Yes |  |
| Bio11 | 0.7086 | 3.64 | - | No | Strong correlation |
| Bio10 | 0.7162 | 2.61 | - | No | Strong correlation |
| Bio16 | 0.7222 | 1.79 | 2 | Yes |  |
| Bio18 | 0.7236 | 1.60 | 3 | Yes |  |
| Bio04 | 0.7249 | 1.43 | 4 | Yes |  |
| Bio19 | 0.7268 | 1.17 | - | No | Strong correlation |
| Bio15 | 0.7285 | 0.94 | - | No | Limited contribution |
| Bio12 | 0.7307 | 0.64 | - | No | Limited contribution + Strong correlation |
| Bio01 | 0.7308 | 0.63 | - | No | Limited contribution + Strong correlation |
| Bio05 | 0.7325 | 0.39 | - | No | Limited contribution |
| Bio13 | 0.7328 | 0.35 | - | No | Limited contribution + Strong correlation |
| Bio03 | 0.7337 | 0.23 | - | No | Limited contribution |
| Bio09 | 0.7337 | 0.23 | - | No | Limited contribution |
| Bio17 | 0.7341 | 0.18 | - | No | Limited contribution + Strong correlation |
| Bio02 | 0.7343 | 0.15 | - | No | Limited contribution + Strong correlation |
| Bio08 | 0.7344 | 0.14 | - | No | Limited contribution + Strong correlation |
| Bio06 | 0.7348 | 0.08 | - | No | Limited contribution + Strong correlation |
| Bio14 | 0.7352 | 0.03 | - | No | Limited contribution + Strong correlation |
| Bio07 | 0.7357 | -0.04 | - | No | Limited contribution + Strong correlation |
