# Supplementary material for: Species distribution models for the eastern blacklegged tick, Ixodes scapularis, and the Lyme disease pathogen, Borrelia burgdorferi, in Ontario, Canada
Source: PLoS One. 2020 Sep 11;15(9):e0238126. doi: 10.1371/journal.pone.0238126 (PMC7485816; doi:10.1371/journal.pone.0238126)
Supplement: S5 Table — (DOCX) [file pone.0238126.s006.docx]

**S5 Table.** **Selection of environmental variables for the *Borrelia burgdorferi* model.**

| ***Borrelia burgdorferi* – Land cover variable selection** | | | | | |
| --- | --- | --- | --- | --- | --- |
|  | **Gain** | **% Decrease in Gain** | **Rank** | **Included in Model** | **Reason(s) Omitted** |
| **Full Model** | **2.4806** |  |  |  |  |
| Water_Prop | 2.3542 | 5.10 | 1 | Yes |  |
| Rural_Prop | 2.3691 | 4.49 | 2 | Yes |  |
| Conif_Prop | 2.3761 | 4.21 | 3 | Yes |  |
| Hedge_Prop | 2.3896 | 3.67 | 4 | Yes |  |
| Conif_Dist | 2.4139 | 2.69 | - | No | Variable already in model |
| Infra_Dist | 2.4203 | 2.43 | 5 | Yes |  |
| Infra_Prop | 2.4224 | 2.35 | - | No | Variable already in model |
| Elevation | 2.4385 | 1.70 | 6 | Yes |  |
| Water_Dist | 2.4450 | 1.44 | - | No | Variable already in model |
| Agri_Prop | 2.4509 | 1.20 | 7 | Yes |  |
| Mixed_Dist | 2.4521 | 1.15 | 8 | Yes |  |
| Sparse_Prop | 2.4585 | 0.89 | - | No | Limited contribution |
| Marsh_Prop | 2.4638 | 0.68 | - | No | Limited contribution |
| Decid_Dist | 2.4668 | 0.56 | - | No | Limited contribution + Strong correlation |
| Agri_Dist | 2.4719 | 0.35 | - | No | Limited contribution + Variable already in model |
| Sparse_Dist | 2.4758 | 0.19 | - | No | Limited contribution |
| Mixed_Prop | 2.4765 | 0.17 | - | No | Limited contribution |
| Hedge_Dist | 2.4783 | 0.09 | - | No | Limited contribution + Variable already in model |
| Marsh_Dist | 2.4788 | 0.07 | - | No | Limited contribution |
| Rural_Dist | 2.4808 | -0.01 | - | No | Limited contribution + Strong correlation |
| Decid_Prop | 2.4812 | -0.02 | - | No | Limited contribution |
|  | | | | | |
| ***Borrelia burgdorferi* – Climate variable selection** | | | | | |
|  | **Gain** | **% Decrease in Gain** | **Rank** | **Included in Model** | **Reason(s) Omitted** |
| **Full Model** | **0.6854** |  |  |  |  |
| Bio10 | 0.6462 | 5.72 | 1 | Yes |  |
| Bio01 | 0.6605 | 3.63 | - | No | Strong correlation |
| Bio19 | 0.6660 | 2.83 | 2 | Yes |  |
| Bio13 | 0.6679 | 2.55 | - | No | Strong correlation |
| Bio11 | 0.6729 | 1.82 | - | No | Strong correlation |
| Bio16 | 0.6735 | 1.74 | - | No | Strong correlation |
| Bio09 | 0.6776 | 1.14 | 3 | Yes |  |
| Bio18 | 0.6784 | 1.02 | 4 | Yes |  |
| Bio04 | 0.6791 | 0.92 | - | No | Limited contribution |
| Bio07 | 0.6797 | 0.83 | - | No | Limited contribution |
| DD>0C | 0.6800 | 0.79 | - | No | Limited contribution + Strong correlation |
| Bio14 | 0.6820 | 0.50 | - | No | Limited contribution + Strong correlation |
| Bio15 | 0.6833 | 0.31 | - | No | Limited contribution |
| Bio05 | 0.6841 | 0.19 | - | No | Limited contribution + Strong correlation |
| Bio03 | 0.6848 | 0.09 | - | No | Limited contribution |
| Bio08 | 0.6851 | 0.04 | - | No | Limited contribution + Strong correlation |
| Bio12 | 0.6852 | 0.03 | - | No | Limited contribution + Strong correlation |
| Bio17 | 0.6852 | 0.03 | - | No | Limited contribution + Strong correlation |
| Bio06 | 0.6853 | 0.01 | - | No | Limited contribution + Strong correlation |
| Bio02 | 0.6854 | 0.00 | - | No | Limited contribution |
